# Inactivation of poly(3-hydroxybutyrate) (PHB) biosynthesis in ‘Knallgas’ bacterium *Xanthobacter* sp. SoF1

**DOI:** 10.1186/s13568-023-01577-0

**Published:** 2023-07-14

**Authors:** Tytti Jämsä, Petri Tervasmäki, Juha-Pekka Pitkänen, Laura Salusjärvi

**Affiliations:** 1 VTT Technical Research Centre of Finland Ltd., 02150 Espoo, Finland; 2 Solar Foods, 53850 Lappeenranta, Finland

**Keywords:** Knallgas, Hydrogen-oxidizing bacteria, Polyhydroxybutyrate, PHB, Polyhydroxyalkanoate, PHA

## Abstract

Aerobic hydrogen-oxidizing ‘Knallgas’ bacteria are promising candidates for microbial cell factories due to their ability to use hydrogen and carbon dioxide as the sole energy and carbon sources, respectively. These bacteria can convert atmospheric CO_2_ to chemicals which could help to mitigate climate change by replacing fossil fuel-based chemicals. A known method to enhance the product yield is to disrupt competing metabolic pathways in the host organism. One such pathway in many ‘Knallgas’ bacteria is polyhydroxybutyrate (PHB) biosynthesis. In this study, the PHB biosynthesis genes of a non-model ‘Knallgas’ bacterium *Xanthobacter *sp. SoF1 were identified. Consequently, the *phaA*, *phaB* and *phaC* genes were individually deleted and the resulting knockouts were evaluated for their ability to produce PHB in autotrophic shake flask and small-scale bioreactor cultivations. The results demonstrate that PHB production was inactivated in the *phaC1* knockout strain, which advances the development of *Xanthobacter *sp. SoF1 as a production host.

**Graphical Abstract:**

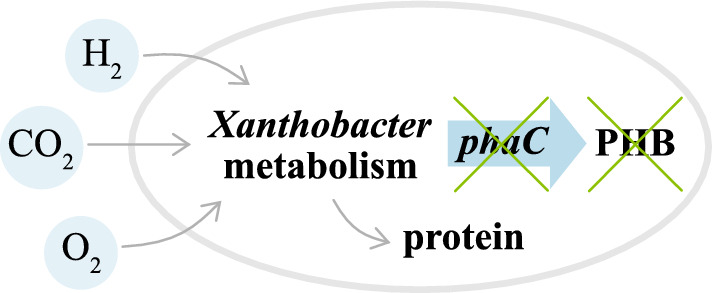

**Supplementary Information:**

The online version contains supplementary material available at 10.1186/s13568-023-01577-0.

## Key points

- *phaA*, *phaB* and *phaC* knockouts of *Xanthobacter *sp. SoF1 were constructed.
- PHB content of the knockouts grown in continuous gas fermentation was determined.
- PHB-deficient *phaC1* knockout strain had higher protein content than the wild type.

**Supplementary Information:**

The online version contains supplementary material available at 10.1186/s13568-023-01577-0.

## Introduction

Current biotechnological production platforms use mainly plant-derived feedstocks, such as sugars and starches, which compete with food production. As an alternative to traditional heterotrophic production hosts, aerobic hydrogen-oxidizing bacteria (HOB), also known as *Knallgas* bacteria, do not require plant-derived feedstocks. They are chemolithoautotrophic organisms using CO_2_ and H_2_ as sole carbon and energy sources, respectively. H_2_ can be generated from water with electrolysis, which can be powered by any renewable energy source. Notably, with solar energy, energy-to-biomass efficiency of *Knallgas* bacteria exceeds that of plants (Liu et al. 2016). At present, various companies, including Air Protein (US), Deep Branch Technology (UK), NovoNutrients (US) and Solar Foods (Finland), develop industrial-scale bioprocesses to cultivate HOB for a high protein content biomass for feed and food purposes (Nyyssölä et al. 2022). When technology for their cultivation and engineering improves, HOB could also become economically feasible production hosts for other biotechnological applications. In fact, prior literature has demonstrated production of organic compounds, such as isobutanol and terpenes, from CO_2_ in engineered HOB, mainly in *Cupriavidus necator* (previously known as *Ralstonia eutropha*) (Brigham 2019). Nevertheless, these studies record low product concentrations that must be increased to reach economically feasible production.

One method to enhance the product yield is to disrupt competing metabolic pathways, such as carbon storage pathways, in the host organism (Funston et al. 2017; Grousseau et al. 2014; van der Woude et al. 2014). In many cases, HOB store carbon and energy in the form of polyhydroxybutyrate (PHB), which accumulates especially under nutrient limitation. For instance, *C. necator*, the model organism for PHB production, can accumulate PHB up to 80% of its cell dry weight (CDW) under autotrophic nitrogen-deficient conditions (Lambauer and Kratzer 2022). In industrial-scale cultivations, nutrient limitation may be avoided by providing cultures with excess nutrients, but this approach lowers the economic feasibility.

PHB biosynthesis requires expression of three genes; *phaA*, *phaB,* and *phaC*, encoding for β-ketothiolase, acetoacetyl-CoA reductase and PHA synthase, respectively. β-ketothiolase joins two acetyl-CoA molecules into acetoacetyl-CoA, which is reduced by acetoacetyl-CoA reductase into 3-hydroxybutyryl-CoA with NAD(P)H as the cofactor. PHA synthase catalyzes the polymerization of 3-hydroxybutyryl-CoA monomers into PHB with the release of CoA. Hence, PHB synthesis consumes one acetyl-CoA and one NAD(P)H for every monomer of PHB, significantly affecting the central metabolism. Redirecting carbon flux from PHB to products in *C. necator* has resulted in the highest product concentrations (isopropanol, 1,3-butanediol and mannitol) recorded for *C. necator* (Garrigues et al. 2020; Gascoyne et al. 2021; Hanko et al. 2022). In these studies, either all three genes related to PHB synthesis or part of them were deleted, depending on the constructed pathway.

Because the vast majority of studies of HOB engineering have been conducted on *C. necator*, the potential of other strains remains mostly unexplored. One potential alternative for *C. necator* is *Xanthobacter *sp. SoF1, which is a non-model, gram-negative, diazotrophic, hydrogen-oxidizing bacterium studied for single cell protein production (Holmström and Pitkänen 2021). Different enzymes (Hirano et al. 2006; Sluis et al. 2002; Zhou et al. 1996) and transcription regulation (van den Bergh et al. 1993; van Keulen et al. 2003) of other *Xanthobacter * spp. have been under investigation. In addition, some genetic engineering tools have been developed for *Xanthobacter * spp. (Larsen et al. 2002; Swaving et al. 1996), which could be adapted for SoF1. However, no prior research has targeted *Xanthobacter *sp. as a host for production of chemicals.

The objective of this study was to inactivate the PHB biosynthesis in SoF1. In the future, this could enable redirection of acetyl-CoA from the PHB synthesis to other products. In addition, genetic engineering methods for targeted integration were established for this novel host. To investigate the effect of PHB pathway genes individually on PHB production, separate *phaA*, *phaB* and *phaC* knockout strains of SoF1 were constructed. The effect of gene knockouts on biomass accumulation, growth rate as well as PHB and protein contents were evaluated in shake flasks and in a continuous gas fermentation under autotrophic nitrogen-limited conditions.

## Materials and methods

### Bacterial strains, culture media and heterotrophic cultivations

The host organism *Xanthobacter * sp. SoF1 (VTT E-193585) was obtained from VTT Culture Collection (Finland). *Escherichia coli* TOP10 was used for plasmid construction. Constructed bacterial strains and plasmids are listed in Table 1. All SoF1 strains were maintained on tryptic soy agar plates (TSA). Cultivations under heterotrophic conditions were carried out in Super Optimal Broth (Hanahan 1983) supplemented with 100 mM ethanol (SOBE) in 100 mL shake flasks in 10 mL cultivation volume at 220 rpm and 30 ºC. Autotrophic cultivation of strains under H_2_–CO_2_–O_2_ was performed in mineral medium DSMZ81 (Deutsche Sammlung von Mikroorganismen und Zellkulturen, http://www.dsmz.de/, Germany) with the following alterations: vanadium, vitamins and sodium bicarbonate were omitted. For shake flask cultivations, nitrogen was supplied as 9 mM of (NH_4_)_2_SO_4_ and for bioreactor cultivations as 16.8 mM of NH_4_OH, if not stated otherwise. In addition, iron was supplied as 0.14 mM of FeSO_4_·7H_2_O for bioreactor cultivations. Cultivation conditions for autotrophic shake flask cultivations and small-scale bioreactors are explained below. Struktol J673A antifoam (Schill + Seilacher, Germany) was added (60 µl/L) to the media for bioreactor cultivations. The media were supplemented with 100 µg/mL of ampicillin, 50 µg/mL of kanamycin or 10 µg/mL of tetracycline, when required.

**Table 1:** Plasmids and bacterial strains used in the study

Plasmid	Description	Reference/source
pUC57	*E. coli* cloning vector with an ampicillin marker	GenBank: Y14837.1
pSF00A	pUC57 with *phaA*::*kan* integration cassette	This study
pSF00B	pUC57 with *phaB*::*kan* integration cassette	This study
pSF00C	pUC57 with *phaC1*::*kan* integration cassette	This study
pSF00D	pUC57 with *phaC2*::*tet* integration cassette	This study
Strain		
*Xanthobacter* sp. SoF1	Wild type	VTT E-193585 (Holmström and Pitkänen 2021)
SoF1Δ*phaA*	SoF1 derivative; *phaA*::*kan*	This study
SoF1Δ*phaB*	SoF1 derivative; *phaB*::*kan*	This study
SoF1Δ*phaC*	SoF1 derivative; *phaC1*::*kan*	This study
SoF1Δ*phaC2*	SoF1 derivative; *phaC2*::*tet*	This study

### Construction of phaA, phaB and phaC knockout strains

Annotation of the whole genome sequence of SoF1 was performed by Biosafe–Biological Safety Solutions Ltd (Kuopio, Finland). A BLAST search was conducted against the genome of SoF1 with *C. necator phaA*, *phaB* and *phaC* genes related to PHB synthesis (GenBank accession no. J04987 and no. J05003) to identify genes for deletion. The genomic DNA of SoF1 was isolated with Easy-DNA gDNA Purification Kit (Invitrogen, USA). Flanking homology arms 1000 bps upstream and downstream of the targeted genes were amplified from the genomic DNA (oligonucleotides presented in Additional file 1: Table S1) with KAPA HiFi DNA polymerase (Kapa Biosystems, USA) using GC buffer. Kanamycin resistance (*kan*) and tetracycline resistance (*tet*) genes, which were placed between the homology arms for selection, were identical to the sequences utilized in plasmids designed for genetic modification of *Xanthobacter flavus* (van den Bergh et al. 1993). Plasmids were constructed into a suicide pUC57 backbone with Gibson assembly using NEBuilder Hifi DNA Assembly Master Mix (NEB, US). Plasmids to target chromosomal homologous recombination were transformed by electroporation into SoF1 cells.

For electrocompetent cells, SoF1 was cultivated in SOBE (10 mL) for 2 days to reach an optical density at 600 nm (OD_600_) of 1–2 as described above. The culture was chilled on ice for 30 min and the cells were harvested by centrifugation at 4 °C and at 3200 g for 20 min and washed with 10 mL of ice-cold water and then with ice-cold 10% glycerol. Cells were resuspended into ice-cold 10% glycerol to reach a concentration of around 2·10^10^ cells/mL and used immediately for electroporation or stored at − 70 ºC.

Cells (50 μL) were mixed with plasmid (~ 100 ng) in a 0.2 cm electroporation cuvette (BTX, US), incubated for 10 min on ice and subjected to a single electric pulse of 2.5 kV with 25 μF capacitance and 600 Ω resistance. Pre-warmed SOBE (1 mL) was added immediately, and the cells were incubated for 4 h at 30ºC before plating on TSA selection plates supplemented with 50 µg/mL of kanamycin or 10 µg/mL of tetracycline and incubated for a minimum of four days in 30 °C. Gene deletion in transformants was confirmed by colony PCR with oligonucleotides listed in Additonal file 1: Table S1. Individual transformants were selected for autotrophic shake flask cultivations.

### Autotrophic shake flask cultivations

Inoculum cultures of SoF1 strains were cultivated heterotrophically as described above. These cultures were used to inoculate modified DSMZ81 media (10 mL) in 100 mL flasks with a plastic cap to attain an initial OD_600_ of 0.5. Three flasks were prepared per strain and grown autotrophically at 30ºC with 136 rpm shaking in a 25.8 L sealed container. Gas flows of 20 mL/min H_2_, 6 mL/min CO_2_ and 3 mL/min O_2_ were fed to the container throughout the cultivation period. The OD_600_ was measured using Spectroquant^®^ Prove 100 (Merck, Germany) every 3 to 4 days, and the ammonia concentration of the supernatants was measured with Ammonia (Rapid) Assay Kit (Megazyme, Ireland). Cultures were cultivated for an additional three to four days after reaching zero ammonia concentration, centrifuged (8 mL) at 4 °C and 10,000g for 30 min, and the cell pellets were lyophilized. The CDWs were determined and PHB contents were analyzed with GC–MS as described by Ylinen et al. (2021).

### Autotrophic small-scale bioreactor cultivations

The comparison of SoF1Δ*phaA*, SoF1Δ*phaB* and SoF1Δ*phaC* strains to the wild type (WT) was performed in small-scale bioreactors (Medicel^®^ Explorer Cultivation Unit, Medicel, Espoo, Finland) under autotrophic conditions. Medicel^®^ Explorer Cultivation Unit is comprised of 15 parallel bioreactors with 230 mL working volume and 5 cm internal diameter. Inoculum cultivations of SoF1 strains for bioreactors were started from glycerol stocks into 10 mL of the modified DSMZ81 media in 250 mL shake flasks with 0.2 μm ePTFE membrane caps. Flasks were incubated in a 34.5 L sealed container in a shaker at 100 rpm under autotrophic conditions (22 mL/min H_2_, 6 mL/min CO_2_ and 3 mL/min O_2_). After 3 days, 50 mL of the same media were added, and incubation was continued for 3 more days.

The precultures were used to inoculate three bioreactors per strain containing 200 mL of media to reach an initial OD_600_ of 0.2. Initially cultivations were fed H_2_–CO_2_–O_2_ gas mixture with 32.5 mL/min H_2_, 6.5 mL/min CO_2_ and 4.5 mL/min O_2_. Based on dissolved oxygen (DO) measurements, the compositions of H_2_–CO_2_–O_2_ gas mixtures were adjusted during the batch phase (Additional file 1: Figures S1-4), after which the following flows per reactor were used in the continuous culture: 32.5 mL/min H_2_, 6.5 mL/min CO_2_ and 1.5–3.5 mL/min O_2_. The gas mixture was fed to the reactors through a sintered sparger and the off-gas flow was diluted with 100 mL/min of N_2_. pH, temperature, and agitation were kept constant at 6.8, 30 °C and 700 rpm, respectively. pH was adjusted with 1 M KOH. The bioreactors were agitated with a magnetic stirrer with an attached flat blade turbine. Sampling (5 mL per reactor) was done using an automatic sampling system once a day and OD_600_ was measured. Before the measurements, samples were kept at 4 °C. Sampling was stopped for 2 days in the batch phase (88–160 h) to conserve cultivation volume. The batch phase of the cultivation was conducted in high ammonia concentration (16.8 mM). In the continuous phase, with a media feed of 2.3 mL/h (dilution rate 0.01 h^−1^), high ammonia concentration (16.8 mM) was used until the steady state was reached, after which the ammonia concentration was decreased to 5.9 mM. Continuous culture was initiated when OD_600_ in a reactor increased above 2.

After cultivation, three 50 mL samples per reactor were taken and centrifuged at 4 °C and 10,000g for 30 min. The cell pellets were lyophilized and their CDWs and PHB contents were determined. In addition, elemental nitrogen content was determined as described previously (Nordlund et al. 2018). Elemental nitrogen values were multiplied by the common conversion factor of 6.25 to estimate the protein content of the cells. Pyruvate concentration of supernatants was measured with high-pressure ion chromatography (HPIC) Dionex ICS-6000 (ThermoFisher Scientific, US).

## Results

### Identification and deletion of genes associated with PHB production in SoF1

**Fig. 1:**
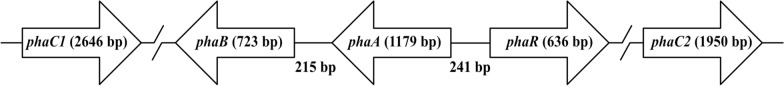
Organization of PHB synthesis genes in the genome of *Xanthobacter *sp. SoF1

The PHB content of the WT SoF1 can reach to 50% or more of its CDW (unpublished data). However, prior to this study, the genes related to PHB synthesis in SoF1 had not been identified. Homologs for *C. necator phaA*, *phaB* and *phaC* genes were searched from the genome of SoF1 using a BLAST search. The search results identified putative *phaA* and *phaB* genes, which possessed 72.3% and 65.7% sequence similarity with the corresponding genes from *C. necator* (GenBank accession no. J04987). These two genes were located in the same operon within the genome of SoF1 together with a plausible *phaR* gene, which is involved in the regulation of PHA synthesis (Maehara et al. 2002). *C. necator phaC* gene (GenBank accession no. J05003) showed 47.6% and 56.2% sequence similarity with two *phaC* genes in SoF1, named here *phaC1* and *phaC2*. Neither of these genes were located in the same operon with *phaA* and *phaB*. Figure 1 illustrates the organization of PHB synthesis-related genes in the genome. The gene sequences can be found from GenBank, using the following accession numbers: OP433461 (*phaA*), OP433462 (*phaB*), OP433463 (*phaC1*), OP433464 (*phaC2*), OP433465 (*phaR*).

In order to inactivate PHB production, SoF1 was transformed with pSF00A-D containing the deletion cassettes for *phaA, phaB, phaC1* and *phaC2* genes of the PHB biosynthetic pathway. After transformation, 11 out of 20 colonies tested had correct gene deletions, which indicated 55% efficiency of targeted integration.

### Comparison of phaA, phaB and phaC knockout strains in shake flasks

**Fig. 2:**
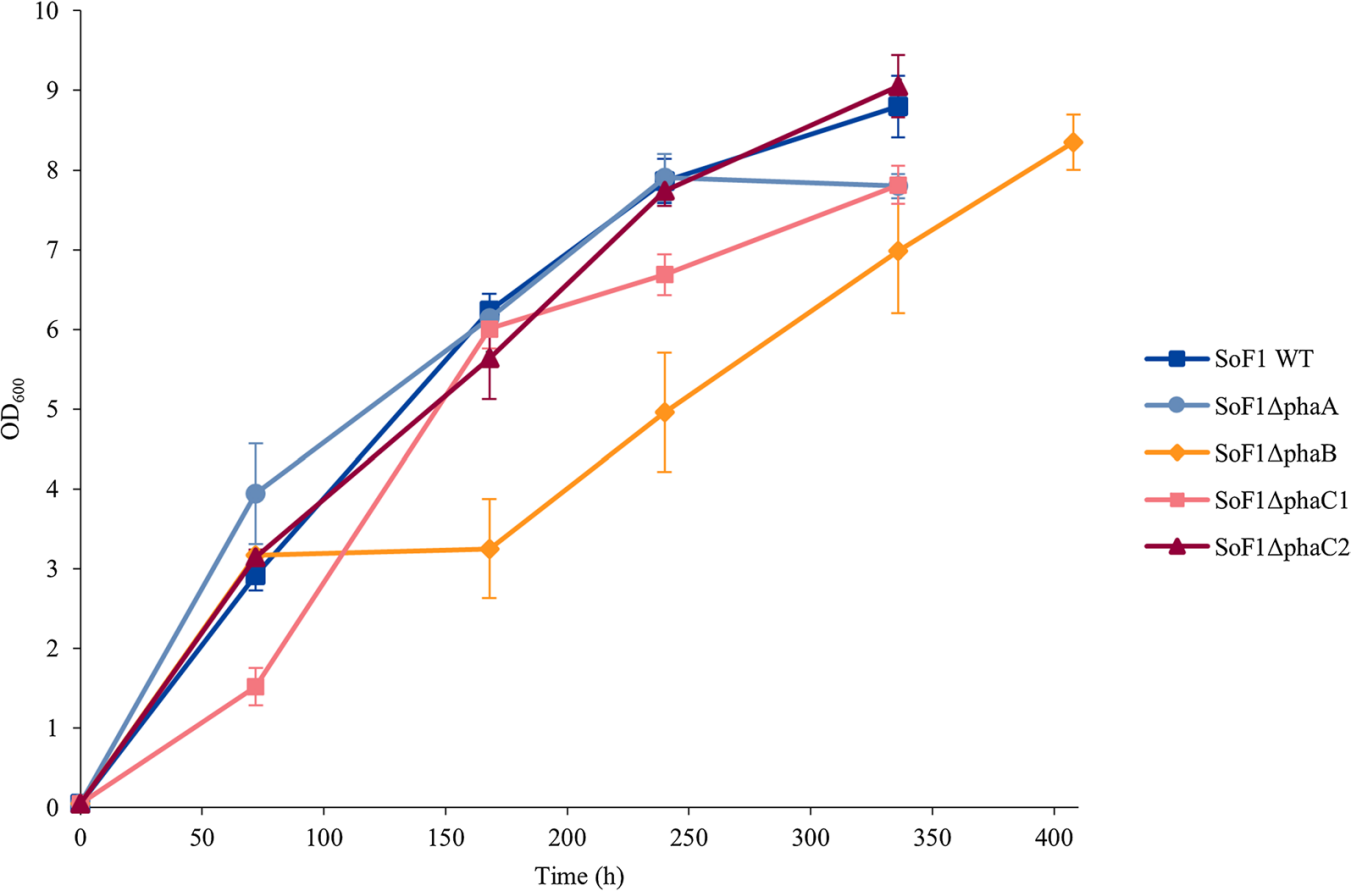
Autotrophic growth of WT SoF1, SoF1Δ*phaA,* SoF1Δ*phaB*, SoF1Δ*pha* and SoF1Δ*phaC2* strains in shake flasks. Results are shown as averages from three parallel shake flask cultivations with error bars representing the standard deviation

The *phaA*, *B* and *C* knockout strains were grown autotrophically in shake flasks to compare their growth rate and PHB accumulation to the WT. The OD_600_ of all strains, except SoF1Δ*phaB* strain, were nearly identical when ammonium was depleted within 240 h (Fig. 2). The ammonium consumption and growth rate of the SoF1Δ*phaB* strain was slower, resulting in a cultivation period being extended by 72 h. However, it eventually reached a similar OD_600_ as the other strains (Fig. 2).

CDW and PHB were quantified from the samples collected at the end of the shake flask cultivations, and the results are presented in Table 2. The deletion of *phaA* gene resulted in a 56% reduction in PHB accumulation. Similarly, PHB production decreased by 90% upon deletion of *phaB* gene. Complete inactivation of PHB synthesis was achieved by knocking-out the *phaC1* gene from the genome. The deletion of *phaC2* did not result in complete inactivation of PHB synthesis but appeared to have a similar effect as *phaA* deletion. Reduced PHB contents in all knockout strains confirmed the involvement of these genes in the PHB biosynthesis in SoF1.

**Table 2:** Effect of deletion of *phaABC* genes from the SoF1 genome on CDW and PHB content of the cells. Cells were grown autotrophically in shake flasks in modified DSMZ81 media. Sampling was performed at the end of the cultivation when ammonia had been depleted for 3 to 4 days. Average from three shake flasks is shown with standard deviation

Strain	OD_600_	CDW (g/L)	PHB (%CDW)
SoF1	8.8 ± 0.4	4.7 ± 0.1	6.3 ± 0.9
SoF1Δ*phaA*	7.8 ± 0.2	3.9 ± 0.1	2.8 ± 0.3
SoF1Δ*phaB*	8.3 ± 0.3	4.6 ± 0.2	0.6 ± 0.1
SoF1Δ*phaC*	7.8 ± 0.2	3.4 ± 0.1	0.0 ± 0.0
SoF1Δ*phaC2*	9.1 ± 0.4	2.6 ± 1.3	2.9 ± 0.7

### Continuous cultivation of phaA, phaB and phaC knockout strains

WT SoF1, SoF1Δ*phaA,* SoF1Δ*phaB* and SoF1Δ*phaC* strains were cultivated in three parallel bioreactors under autotrophic conditions. SoF1Δ*phaC2* was excluded from these cultivations because its PHB synthesis was not as strongly inhibited as in SoF1Δ*phaC* strain. The bioreactor cultivations were initiated in high ammonium concentration (16.8 mM) as batch cultivations in order to accumulate biomass. Continuous cultivation was started when OD_600_ reached 2. Continuous cultivation at a constant dilution rate allowed the comparison of the knockout strains at a steady state with a constant growth rate of 0.01 h^−1^. At the end of the continuous cultivation (304 h) the ammonium concentration was lowered to 5.9 mM to induce PHB production further. To confirm the nitrogen-limitation, ammonium was measured from the culture supernatants at time points 232 h, 256 h and 400 h. The initial gas flows were the same for all strains but later on the inlet CO_2_:O_2_:H_2_ gas ratio was adjusted for each reactor depending on the dissolved oxygen (DO). Steady increase in DO, meaning that all oxygen was not consumed, indicated slow growth and possibly oxygen-intolerance. The DO data and used gas compositions for all the bioreactors are presented in Additional file 1: Figures S1–4.

**Fig. 3:**
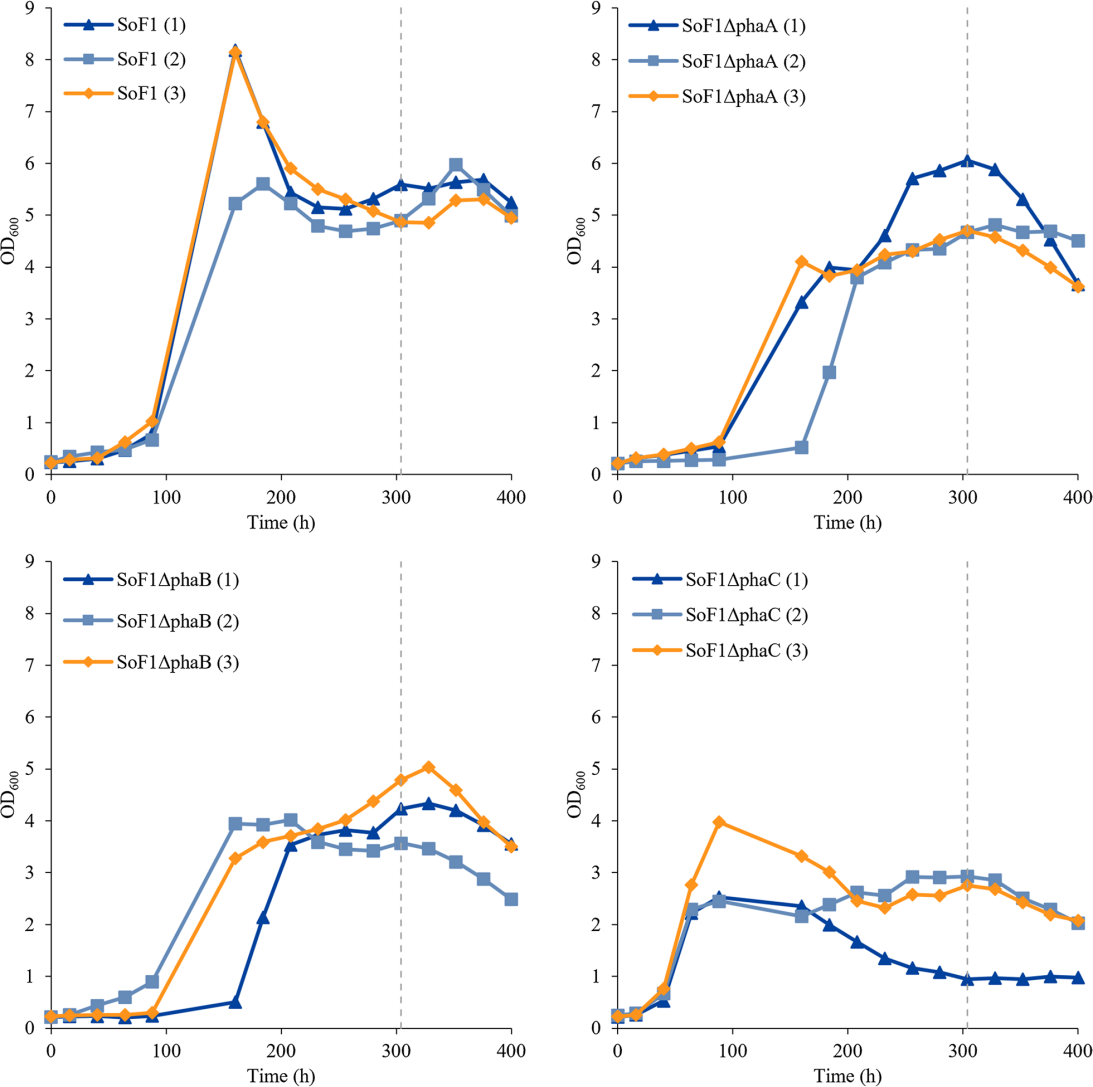
Autotrophic growth of WT SoF1, SoF1Δ*phaA, B* and *C* strains in 12 small-scale bioreactors. The batch phase was conducted in modified DSMZ81 media with high nitrogen concentration (16.8 mM of NH_4_OH). Continuous phase was started in reactors when OD_600_ was above 2. Same media as in batch phase was applied for continuous culture at a dilution rate of 0.01 h^−1^ until low nitrogen concentration (5.9 mM of NH_4_OH) feed was started at 304 h (dashed line)

Figure 3 presents the growth curves for WT SoF1, SoF1Δ*phaA*, *B* and *C* in three parallel bioreactors. SoF1Δ*phaC* had the shortest lag phase in the batch phase; OD_600_ in all three SoF1Δ*phaC* reactors was above 2 at 64 h whereas at the same time point other strains had OD_600_ below 1. In the batch phase, the growth of individual strains in separate bioreactors was comparable, except for one outlier bioreactor for each strain. However, more variation was observed in the continuous cultivation. This might be caused by the long duration of the cultivation highlighting the possible differences between the individual reactor vessels such as agitation efficiency and gas diffusion.

**Fig. 4:**
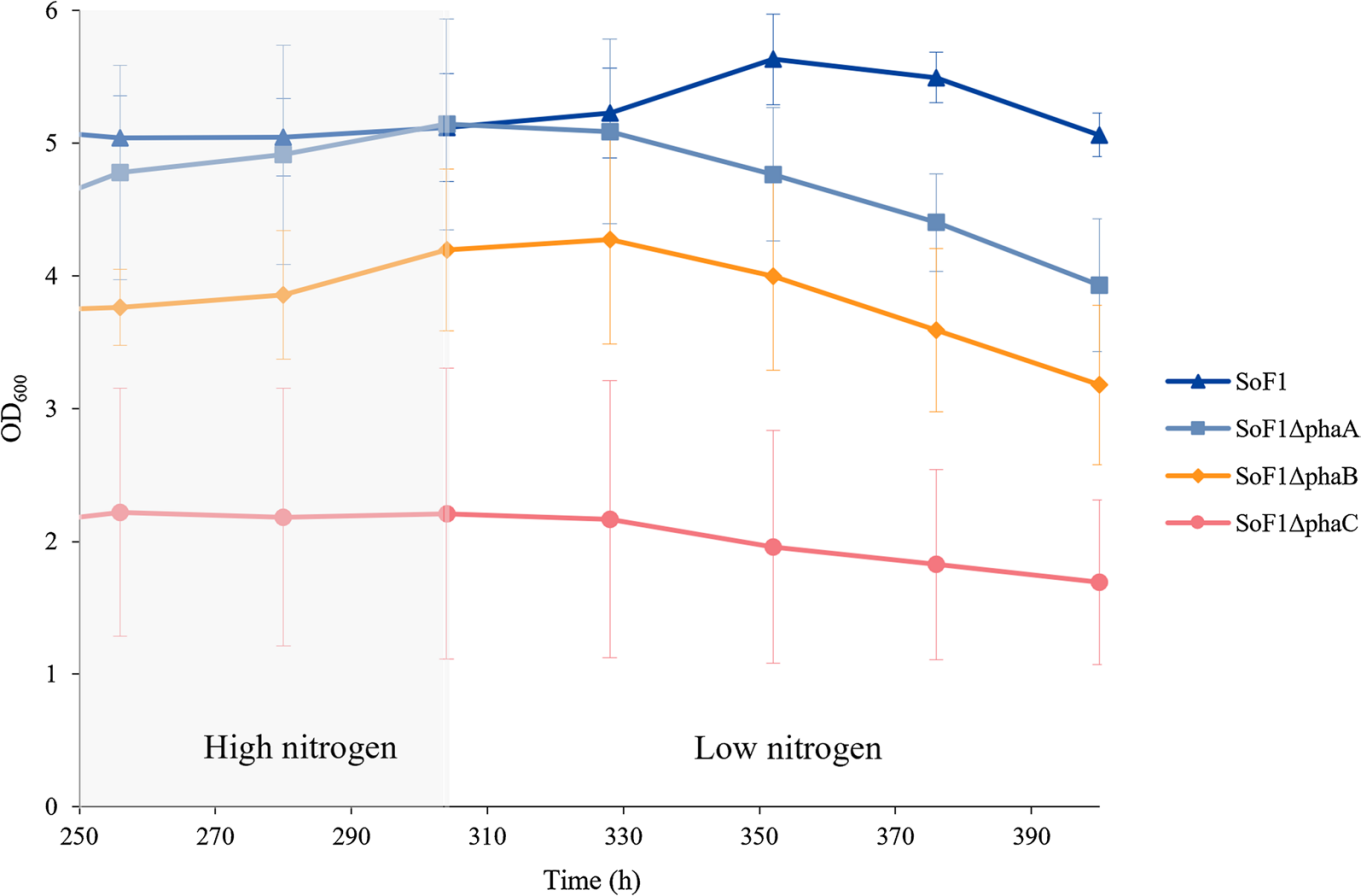
Growth of SoF1, SoF1Δ*phaA, B* and *C* strains in autotrophic continuous cultivation at high nitrogen (16.8 mM of NH_4_OH) and low nitrogen (5.9 mM of NH_4_OH) concentrations. Results are shown as averages of three parallel bioreactor cultivations with error bars representing the standard deviation

To illustrate the differences in the continuous cultivation between the constructed strains and WT SoF1, Fig. 4 represents the average growth curves of the strains when steady state was reached in continuous cultures and after low nitrogen concentration was applied. All strains had higher OD_600_ during the high nitrogen concentration (16.8 mM of NH_4_OH) and OD_600_ decreased when lower concentration was applied (5.9 mM of NH_4_OH). In both cases, the measured ammonia from the supernatant was close to zero (time points 232 h, 256 h and 400 h). At a high nitrogen concentration, OD_600_ of WT SoF1 settled to around 5 while the biomass accumulation rate decreased with gene deletions in subsequent steps along the PHB pathway. SoF1Δ*phaA* had slightly lower OD_600_ compared to WT SoF1 during the high nitrogen phase of the continuous cultivation but its OD_600_ decreased more during the low nitrogen phase. OD_600_ of SoF1Δ*phaB* settled around 4 during the high nitrogen phase and decreased to 3 during the low nitrogen phase. SoF1Δ*phaC* reached only OD_600_ of 2 with a slight decrease when low nitrogen was applied. The differences in parallel cultivations for each strain resulted in high standard deviations from average growth. However, the results indicate that SoF1Δ*phaC* accumulated less biomass in the continuous phase than the other strains.

**Fig. 5:**
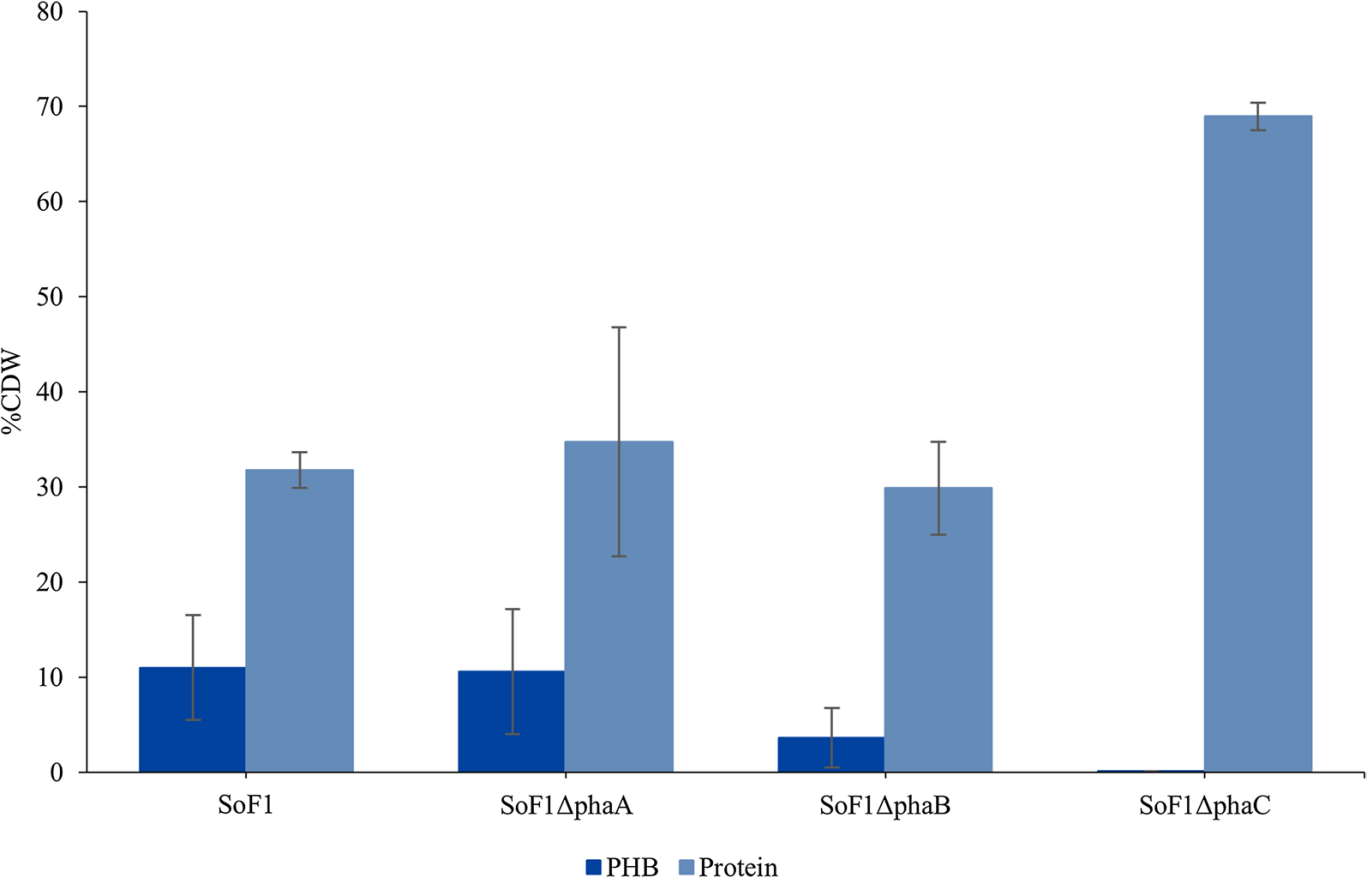
Effect of deletion of *phaABC* genes from the SoF1 genome on PHB and protein contents of cells grown in an autotrophic continuous cultivation. Cells were cultivated in modified DSMZ81 media for 400 h and sampling was performed at the end of the cultivation. Average from three parallel cultivations is shown with standard deviation

Samples were taken at the end of the continuous cultivations to analyze PHB and protein contents of the strains (Fig. 5). The results for each separate cultivation are presented in Additional file 1: Table S2. Because bioreactor cultivations can be sensitive to the slightest differences in culture conditions, such as above-mentioned agitation efficiency and gas diffusion, the differences in produced PHB of separate bioreactors of each studied strain were substantial. However, a similar trend can be seen as in shake flask cultivations: deletion of *phaB* inhibited PHB production more than *phaA* deletion. In alignment with shake flask cultivations, the strain with *phaC* deletion did not produce PHB. The analysis of elemental nitrogen content of the cells revealed that the PHB-deficient strain accumulated approximately twice the amount of protein compared to the other strains. The hypothesis was that the reduced PHB synthesis could also result in excretion of pyruvate, but no pyruvate was detected from the supernatants.

## Discussion

Using HOB production hosts in biotechnology applications could enable carbon–neutral or even carbon-negative production of chemicals, feed, and food by using CO_2_ from the atmosphere and green H_2_ as a feedstock. This study took the first steps in developing one potential production host, *Xanthobacter *sp. SoF1, towards this goal by identifying and individually deleting its PHB biosynthesis genes *phaA*, *phaB* and *phaC*. The objective of inactivating PHB synthesis was achieved in the SoF1Δ*phaC* knockout strain. To the best of our knowledge, this is the first time that PHB-related genes have been experimentally verified in *Xanthobacter *sp.

The deletion of *phaA* and *phaB* genes in SoF1 did not fully disrupt PHB synthesis. The same was observed in *C. necator phaA* and *phaB* knockouts, which retain their ability to produce PHB because of the presence of other isoenzymes (Budde et al. 2010; Lindenkamp et al. 2010). *C. necator* has at least 20 other β-ketothiolases that can assist in PHB production when *phaA* is deleted (Arikawa and Sato 2022). A BLAST search conducted on the SoF1 genome with the *C. necator phaA* gene as the query identified multiple closely related genes in addition to the highly related *phaA*, which was knocked out in this study. Regarding *phaB* homologs in *C. necator*, Budde et al. (2010) identified three, which they targeted for deletion. Out of these genes, one (*phaB2*) was not expressed as an active acetoacetyl-CoA reductase, but the deletion of the other two inactivated PHB synthesis. Although the BLAST query coverages in SoF1 for other than the deleted *phaB* gene were low (< 33%), it is likely that SoF1 also possesses other enzymes with acetoacetyl-CoA reductase activity.

Based on previous literature, it was expected that a *phaC* knockout of SoF1 would not produce PHB. Although some organisms have more than one functional *phaC* gene (Rehm and Steinbüchel 1999), deletion of only one gene, *phaC1*, from *C. necator* has been shown to prevent PHB synthesis (Park et al. 2010). *Cupriavidus necator* possesses a second PHA synthase PhaC2 which is unexpressed, at least under the tested conditions (Peplinski et al. 2010). The present study shows that also SoF1 possesses one PHA synthase, which deletion is enough to disrupt the PHB biosynthesis. However, the role of *phaC2* gene is unclear because its deletion lowered PHB production but did not contribute to PHB production in SoF1Δ*phaC* strain. PHB biosynthesis in other HOB than *C. necator* has remained quite unexplored. However, similar results have also been obtained from *Rhodobacter capsulatus*, a phototrophic non-sulfur purple bacteria that can grow chemolithoautotrophically under aerobic conditions (Paoli and Tabita 1998). Deletion of *phaAB* genes from *R. capsulatus* did not reduce PHB production, while deletion of *phaC* led to complete disruption of it (Kranz et al. 1997). As in SoF1, *phaC* gene of *R. capsulatus* is separate from the *phaAB* operon.

Initially, the knockout strains were investigated in shake flasks, which provided evidence of PHB deficiency. Continuous cultivation was then applied to further analyze the strains at a constant growth rate. Biomass was first accumulated after which PHB accumulation was induced, which is a common strategy in biotechnology. Garcia-Gonzalez et al. (2015) investigated PHB production in *C. necator* with a two-phase cultivation system in which they first accumulated biomass under heterotrophic conditions, after which nitrogen- and oxygen-limiting conditions were applied in the autotrophic phase to induce PHB production. In the PHB accumulation phase, CDW doubled in some reactors solely due to PHB. In this study, indications on the opposite trend were observed when OD_600_ values decreased under low nitrogen concentration in the continuous cultivation. However, ammonium was completely consumed by the cells also in the high nitrogen concentration medium and it might be that the used low nitrogen condition was excessive for the cells. It has been shown that stress response, for example caused by too high salt concentration, in *C. necator* can lead to PHB degradation (Obruca et al. 2010). If this is the case and PHB was produced already in the cultivation phase with high nitrogen concentration, higher PHB concentrations would have been recorded, if cultivations had been stopped earlier. Unfortunately, determination of PHB contents during the cultivation was not possible because of limited sample volumes.

The results from bioreactor cultivations suggest oxygen-sensitivity of the SoF1 strains. In the beginning of the cultivation, all strains except SoF1Δ*phaC* had increasing DO. When oxygen was lowered from 10% of total gas flow to 6%, the growth was retrieved. Oxygen might have affected the activity of hydrogenase and ribulose-1,5-bisphosphate carboxylase/oxygenase (rubisco), which are both crucial for autotrophic growth. In particular, hydrogenases provide cells energy to perform CO_2_ fixation by rubisco. Although HOB are recognized by the oxygen-tolerant hydrogenases, they can be inhibited by high oxygen concentration. Wilde and Schlegel (1982) evaluated the growth of *C. necator* and *Xanthobacter autotrophicus* in autotrophic conditions under different oxygen concentrations (10–100%). The growth rates and hydrogenase activities declined by increasing oxygen concentration but retrieved after returning to a lower oxygen concentration. On the other hand, high O_2_ concentration can induce the oxygenation side reaction of rubisco (Tcherkez 2016). This is less likely to affect the growth of SoF1 because the concentration of CO_2_ was high, which favors carboxylation over oxygenation, especially when considering the lower solubility of O_2_ to media compared to CO_2_. In the future, cultivations could be initiated with a lower oxygen concentration and increased when more biomass is accumulated.

Growth rate and biomass accumulation are frequently affected by genetic modifications. The hypothesis was that knocking-out *phaA* and *phaB* genes earlier from the PHB pathway would prevent accumulation of intermediate compounds, acetoacetyl-CoA and 3-hydroxybutyryl-CoA, possibly impairing growth. Although the growth rate of the knockout strains was not notably impaired in shake flasks in this study, lower biomass accumulation was detected for all knockout strains in the continuous cultivation, especially for SoF1Δ*phaC* strain. Similarly, *Burkholderia thailandesis phaC* knockout strain accumulated 70% less biomass than the WT after 264 h fermentation (Funston et al. 2017), which is comparable to the 80% reduction in biomass of SoF1Δ*phaC* observed in this study. At least two factors might contribute to this outcome. Firstly, it might be that *phaC* knockouts accumulate PHB synthesis intermediate compound 3-HB-CoA inside the cells whereas, for example, acetoacetyl-CoA possibly accumulating when *phaB* is deleted can be more easily redirected to other pathways. This could be avoided by deleting all PHB genes simultaneously in SoF1. Deletion of *phaC* in *C. necator* decreases growth rate, even in shake flasks (Park et al. 2010), but *phaCAB* knockout strain has been shown to accumulate biomass similarly to the WT (Lu et al. 2012). Secondly, the lack of PHB granules results in losing their beneficial functions in protecting against various stress factors (Obruca et al. 2020) and helping with redox imbalance (Batista et al. 2018). This could be possibly avoided by better process control in bioreactors.

A PHB deficient strain of *C. necator* is typically chosen as a production host because it accumulates pyruvate (Lu et al. 2012; Steinbüchel and Schlegel 1989), which can be redirected to other compounds when PHB biosynthesis is blocked. In addition to *phaC* deletion, deletion or overexpression of *phaA* and *phaB* may be needed if precursors are derived from acetoacetyl-CoA or 3-hydroxybutyryl-CoA intermediates of the PHB pathway (Gascoyne et al. 2021). However, studies in which the product was not directly derived from PHB pathway intermediates, show contradicting results on the benefits of this approach. Chen et al. (2015) demonstrated that production of laureate (medium-chain fatty acid) in *C. necator* was not enhanced by *phaCAB* deletion compared to the WT. Similar outcome was found when producing methyl ketones (Muller et al. 2013). On the contrary, production of mannitol was increased by 25% in *C. necator phaCAB* knockout compared to the PHB producing strain (Hanko et al. 2022). Therefore, it seems to differ from case to case if a constructed pathway benefits from PHB-deficiency. In the current study, pyruvate could not be detected from the cultivation supernatants of the PHB-deficient strain. However, the results suggest that the available carbon was possibly directed towards the production of protein rather than pyruvate, and therefore PHB-deficiency is a viable approach to advance SoF1 as a production host for single cell protein. To conclude, the current study took an essential step towards redirecting the carbon flux of SoF1 from PHB biosynthesis to desired products. By enabling further engineering of SoF1, this study unlocks new possibilities for sustainable bioproduction.

## Supplementary Information

** Additional file 1: Fig. S1. **Optical density at 600 nm (OD600) and normalized dissolved oxygen (DO) of three parallel autotrophic bioreactor cultivations of wild-type *Xanthobacter *sp. SoF1 with used gas flows (mL/min). Continuous culture was started at 160 h. **Fig. S2. **OD600 and normalized DO of three parallel autotrophic bioreactor cultivations of *Xanthobacter *sp. SoF1Δ*phaA *with used gas flows (mL/min). Continuous culture was started at 160 h for reactors 1 and 3 and at 184 h for reactor 2. **Fig. S3. **OD600 and normalized DO of three parallel autotrophic bioreactor cultivations of *Xanthobacter *sp. SoF1Δ*phaB *with used gas flows (mL/min). Continuous culture was started at 160 h for reactors 2 and 3 and at 184 h for reactor 1. **Fig. S4. **OD600 and normalized DO of three parallel autotrophic bioreactor cultivations of *Xanthobacter *sp. SoF1Δ*phaC *with used gas flows (mL/min). Continuous culture was started at 64 h. **Table S1. **Oligonucleotides used in the study. **Table S2. **Effect of deletion of *phaABC *genes from the SoF1 genome on CDW, PHB and protein contents of cells grown in an autotrophic continuous cultivation. Cells were cultivated in modified DSMZ81 media for 400 h and sampling was performed at the end of the cultivation. Average from three parallel cultivations is shown with standard deviation (std).

## Data Availability

All relevant data are included in this article and its Additional file 1.

## Abbreviations

CDW: Cell dry weight
DO: Dissolved oxygen
HOB: Hydrogen-oxidizing bacteria
PHB: Polyhydroxybutyrate
SOBE: Super optimal broth supplemented with 100 mM of ethanol
TSA: Tryptic soy agar
WT: Wild type
