# Supplementary material for: Inactivation of poly(3-hydroxybutyrate) (PHB) biosynthesis in ‘Knallgas’ bacterium Xanthobacter sp. SoF1
Source: AMB Express. 2023 Jul 14;13:75. doi: 10.1186/s13568-023-01577-0 (PMC10349022; doi:10.1186/s13568-023-01577-0)
Supplement: Supplementary file 1 — Additional file 1: Fig. S1. Optical density at 600 nm (OD600) and normalized dissolved oxygen (DO) of three parallel autotrophic bioreactor cultivations of wild-type Xanthobacter sp. SoF1 with used gas flows (mL/min). Continuous culture was started at 160 h. Fig. S2. OD600 and normalized DO of three parallel autotrophic bioreactor cultivations of Xanthobacter sp. SoF1ΔphaA with used gas flows (mL/min). Continuous culture was started at 160 h for reactors 1 and 3 and at 184 h for reactor 2. Fig. S3. OD600 and normalized DO of three parallel autotrophic bioreactor cultivations of Xanthobacter sp. SoF1ΔphaB with used gas flows (mL/min). Continuous culture was started at 160 h for reactors 2 and 3 and at 184 h for reactor 1. Fig. S4. OD600 and normalized DO of three parallel autotrophic bioreactor cultivations of Xanthobacter sp. SoF1ΔphaC with used gas flows (mL/min). Continuous culture was started at 64 h. Table S1. Oligonucleotides used in the study. Table S2. Effect of deletion of phaABC genes from the SoF1 genome on CDW, PHB and protein contents of cells grown in an autotrophic continuous cultivation. Cells were cultivated in modified DSMZ81 media for 400 h and sampling was performed at the end of the cultivation. Average from three parallel cultivations is shown with standard deviation (std). [file 13568_2023_1577_MOESM1_ESM.pdf]

AMB Express

Supplementary material for:

Inactivation of poly(3-hydroxybutyrate) (PHB) biosynthesis in ‘Knallgas’ bacterium *Xanthobacter sp.* SoF1

Tytti Jämsä[1], Petri Tervasmäki[2], Juha-Pekka Pitkänen[2], Laura Salusjärvi[1]

[1] VTT Technical Research Centre of Finland Ltd., 02150 Espoo, Finland
[2] Solar Foods, 53850 Lappeenranta, Finland

Corresponding author: Tytti Jämsä

tytti.jamsa@vtt.fi

Supplementary Table S1 Oligonucleotides used in the study

**Name**	**Sequence**
Δ*phaA* downstream F	GTCACGACGTTGTAAAACGACGGCCAGTGAATTCGAGCTTTCACGGTCATGGTGGAGC
Δ*phaA* downstream R	GTCCAGATAGCCCAGTAGCTGACATTCATCCGGGGTCATCCAAGGTGAACGTGAACGG
Δ*phaA* upstream F	AATTGCAGTTTCATTTGATGCTCGATGAGTTTTTCTAAACAGGCCGTCCTTGATCATG
Δ*phaA* upstream R	TTGCATGCAGGCCTCTGCAGTCGACGGGCCCGGTTAATCTTCGTCATATGGTCGCGGA
Δ*phaB* downstream F	TCACGACGTTGTAAAACGACGGCCAGTGAATTCGAGCTCGACATCCTCTCCGACATCG
Δ*phaB* downstream R	GTCCAGATAGCCCAGTAGCTGACATTCATCCGGGGTCACGCCCAGTACATCACCTGAG
Δ*phaB* upstream F	AATTGCAGTTTCATTTGATGCTCGATGAGTTTTTCTAACCTTCAACCCCACCGAGATC
Δ*phaB* upstream R	TTGCATGCAGGCCTCTGCAGTCGACGGGCCCGGTTAATATGATCAAGGACGGCCTGTG
Δ*phaC1* downstream F	AGGCTGAAAAGCCGGCCCCCGCTGCGGCCCTTAATTAACCCGCAGGATGGGGTGGAGC
Δ*phaC1* downstream R	AGCCCAGTAGCTGACATTCATCCGGGGTCAATCGGCGACCCAGTATTCCA
Δ*phaC1* upstream F	TTTCATTTGATGCTCGATGAGTTTTTCTAAAATCTGGTGAAGGGCATGCA
Δ*phaC1* upstream R	CATGCAGGCCTCTGCAGTCGACGGGCCCGGTTAATTAACTTCATCTTGGCCGGCGGGT
Δ*phaC2* downstream F	GGCAAGGCTGAAAAGCCGGCCCCCGCTGCGGCCCTTAATGTCCGATCTGCCCGTCAT
Δ*phaC2* downstream R	TGCAGCACTGGGGCCAGATGGTAAGCCCTCCCGTATCGTAAGATGCATCGCCCAATCC
Δ*phaC2* upstream F	CTGTAAGCGGATGCCGGGAGCAGACAAGAGACGCTAGGGGCCAATTTCGTCCAGGGCG
Δ*phaC2* upstream R	GCTTGCATGCAGGCCTCTGCAGTCGACGGGCCCGGTTAATTGGTGTCGTGGATGCGAT
kan F	TGCCTCGGTGAGTTTTCTCC
kan R	TAAGCCCACTGCAAGCTACC
tet F	CAGACGGTCACAGCTTGTCT
tet R	CACTGGGGCCAGATGGTAAG
*phaA*locus F	GCCTCAGGTGATGTACTGGG
*phaA*locus R	TCTCTGTCTTTGGGAACGGC
*phaB*locus F	GAGCACAACTTGGCCGATTC
*phaB*locus R	AATCCATGAGCCAGTCCACC
*phaC1*locus F	CCTCACATCCCCCAATGTCC
*phaC1*locus R	ACCTGGTGGACCGTTCCT
*phaC2*locus F	GTTCAGCATGGGGTTGGAGA
*phaC2*locus R	CGCTGGAAAAGGCCAATGAG

Supplementary Table S2 Effect of deletion of *phaABC* genes from the SoF1 genome on CDW, PHB and protein contents of cells grown in an autotrophic continuous cultivation. Cells were cultivated in modified DSMZ81 media for 400 h and sampling was performed at the end of the cultivation. Average from three parallel cultivations is shown with standard deviation (std)

**Strain (reactor #)**	**OD_{600}**	**CDW (g/L)**	**PHB (%CDW)**	**Elemental nitrogen (%CDW)**	**Protein (%CDW)**
SoF1 (1)	5.3	2.9	5.6	4.8	30.3
SoF1 (2)	5.0	3.3	8.8	4.9	30.5
SoF1 (3)	5.0	2.7	18.6	5.5	34.4
Average±std	5.1±0.1	3.0±0.3	11.0±5.5	5.1±0.3	31.8±1.9
SoF1Δ*phaA* (1)	3.7	1.2	6.2	8.1	50.4
SoF1Δ*phaA* (2)	4.5	2.2	19.9	3.4	21.0
SoF1Δ*phaA* (3)	3.6	2.2	5.6	5.2	32.8
Average±std	3.9±0.4	1.8±0.5	10.6±6.6	5.6±1.9	34.7±12.1
SoF1Δ*phaB* (1)	3.6	2.3	7.9	4.6	28.7
SoF1Δ*phaB* (2)	2.5	1.5	0.6	3.9	24.5
SoF1Δ*phaB* (3)	3.5	2.2	2.4	5.8	36.4
Average±std	3.2±0.5	2.0±0.4	3.6±3.1	4.8±0.8	29.9±4.9
SoF1Δ*phaC* (1)	1.0	0.3	0.0	10.7	66.9
SoF1Δ*phaC* (2)	2.0	0.6	0.0	11.2	70.0
SoF1Δ*phaC* (3)	2.1	0.6	0.2	11.2	69.9
Average±std	1.7±0.5	0.5±0.1	0.1±0.1	11.0±0.2	69.0±1.4

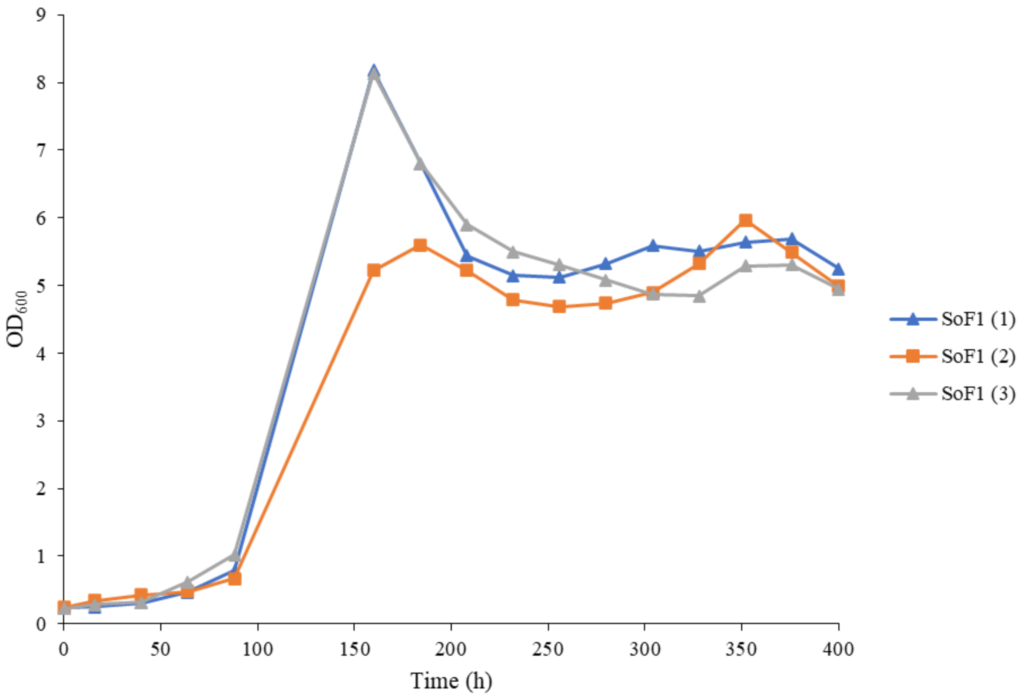

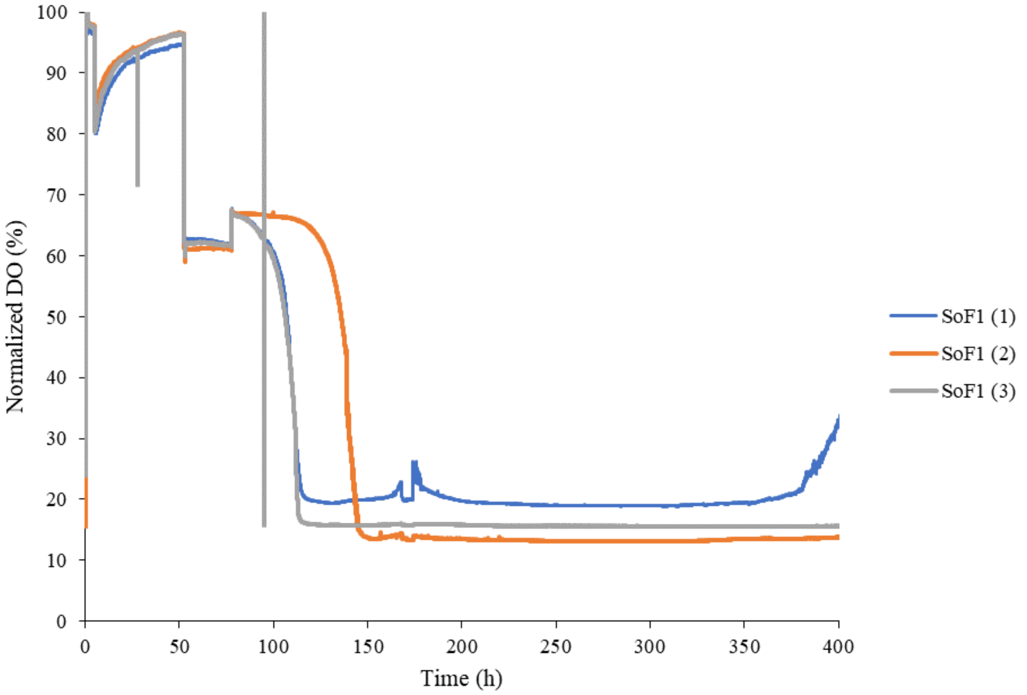

**Time (h)**	**0**	**52.5**	**77.83**	**174**
H_2	32.5	32.5	26.3	32.5
CO_2	6.5	6.5	7	6.5
O2	4.5	2.5	2.5	3.5

Fig. S1 Optical density at 600 nm (OD_{600}) and normalized dissolved oxygen (DO) of three parallel autotrophic bioreactor cultivations of wild-type *Xanthobacter sp.* SoF1 with used gas flows (ml/min). Continuous culture was started at 160 h

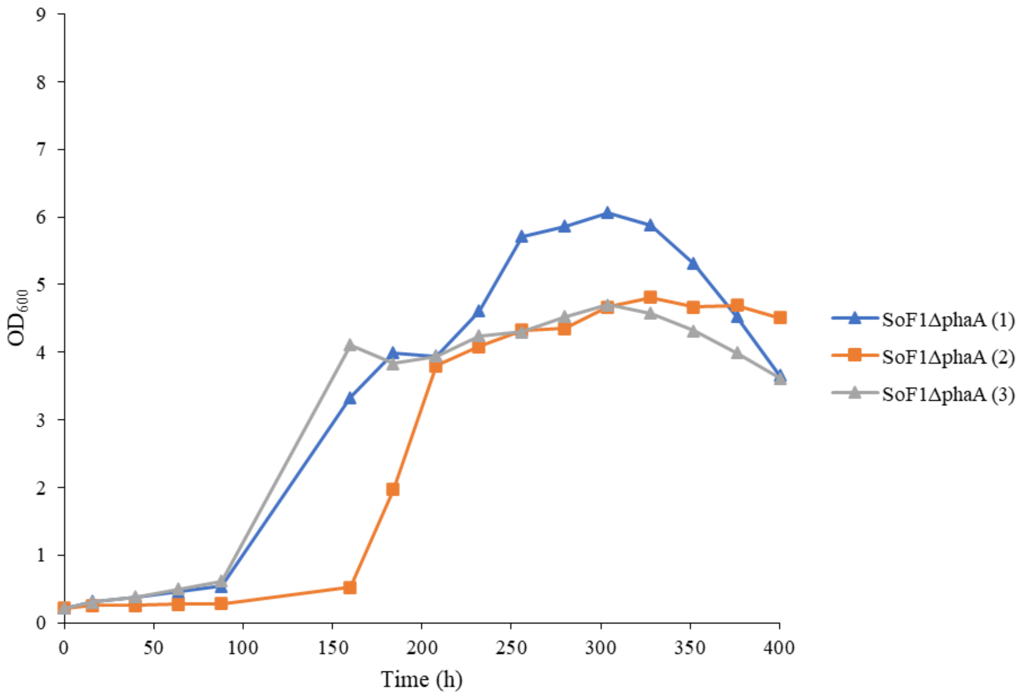

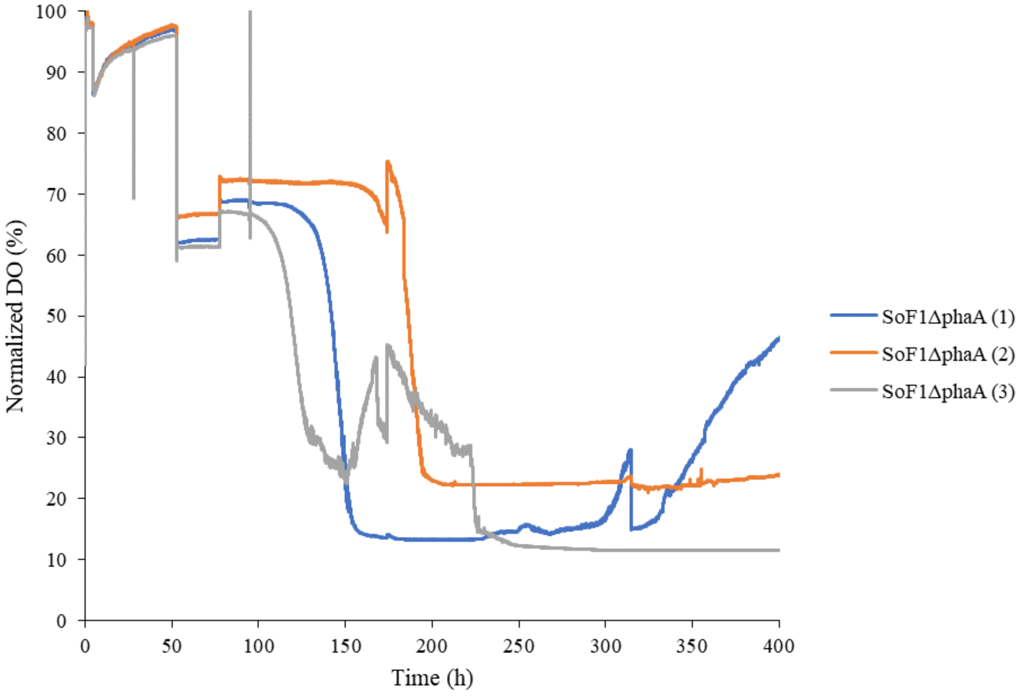

**Time (h)**	**0**	**52.5**	**77.83**	**174**	**314.5 (1)**
H_2	32.5	32.5	26.3	32.5	32.5
CO_2	6.5	6.5	7	6.5	6.5
O_2	4.5	2.5	2.5	3.5	2.5

Fig. S2 OD_{600} and normalized DO of three parallel autotrophic bioreactor cultivations of *Xanthobacter sp.* SoF1Δ*phaA* with used gas flows (ml/min). Continuous culture was started at 160 h for reactors 1 and 3 and at 184 h for reactor 2

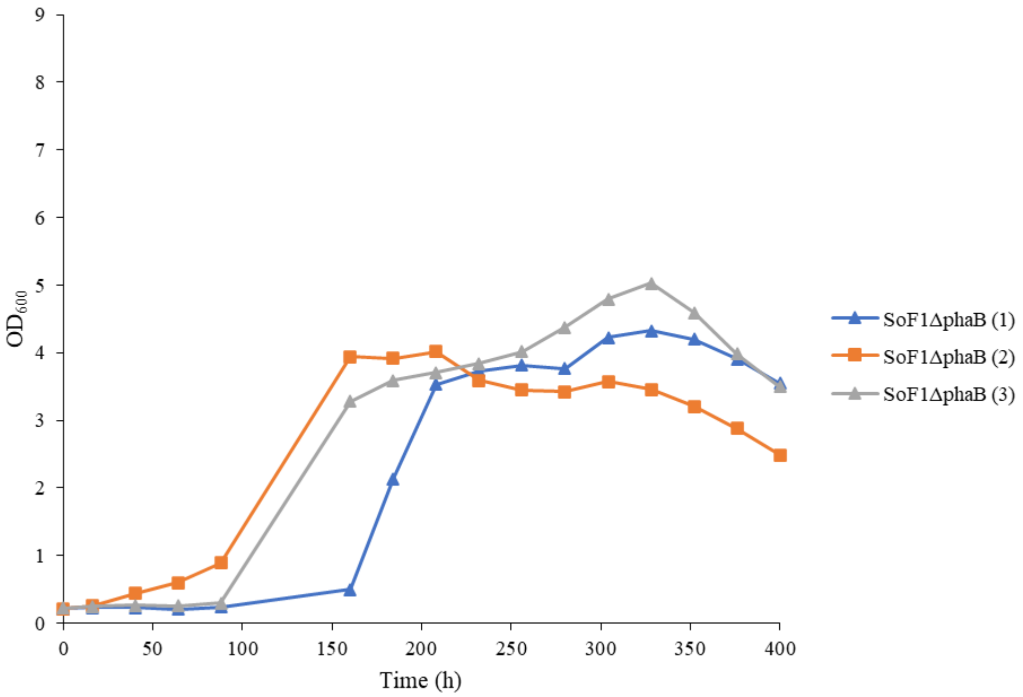

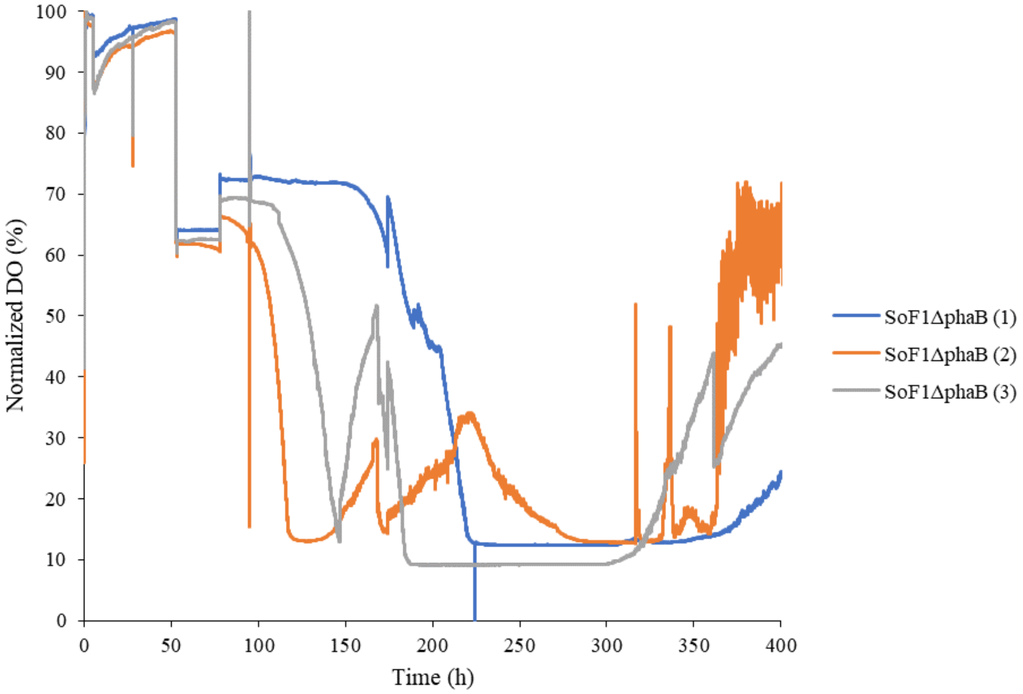

**Time (h)**	**0**	**52.5**	**77.83**	**174**	**361.5 (3)**
H_2	32.5	32.5	26.3	32.5	32.5
CO_2	6.5	6.5	7	6.5	6.5
O_2	4.5	2.5	2.5	3.5	2.5

Fig. S3 OD_{600} and normalized DO of three parallel autotrophic bioreactor cultivations of *Xanthobacter sp.* SoF1Δ*phaB* with used gas flows (ml/min). Continuous culture was started at 160 h for reactors 2 and 3 and at 184 h for reactor 1

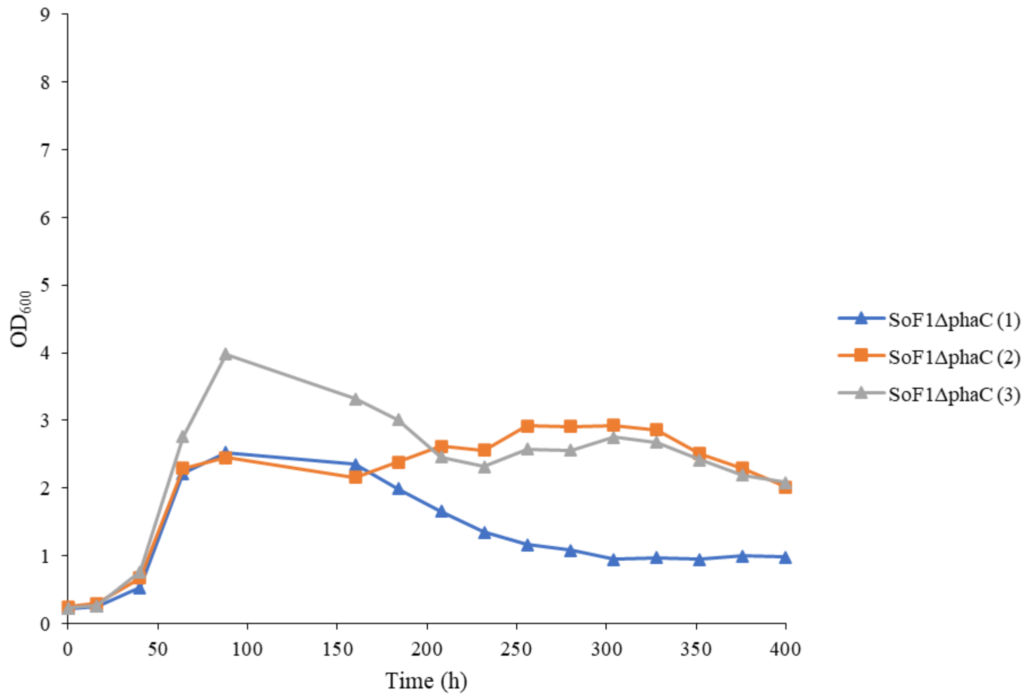

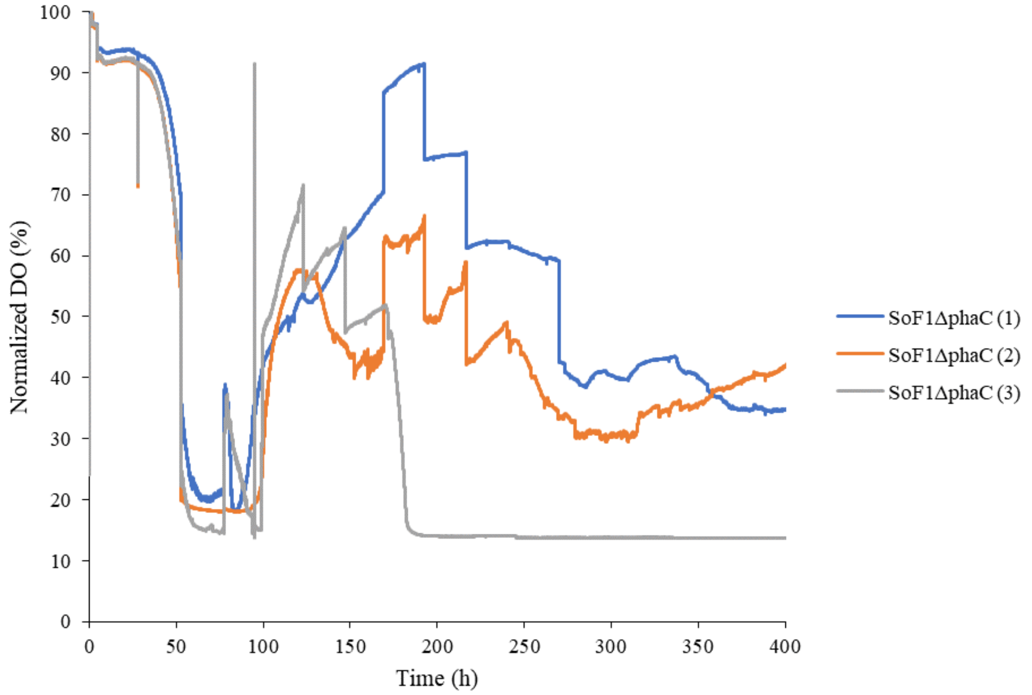

**Time (h)**	**0**	**52.5**	**77.83**	**123 (3)**	**146 (3)**	**169**	**192.5**	**216.5**	**270 (1)**
H_2	32.5	32.5	32.5	32.5	32.5	32.5	32.5	32.5	32.5
CO_2	6.5	6.5	6.5	6.5	6.5	6.5	6.5	6.5	8
O_2	4.5	2.5	3.5	2.5	1.5	4.5	3.5	2.5	1.5

Fig. S4 OD_{600} and normalized DO of three parallel autotrophic bioreactor cultivations of *Xanthobacter sp.* SoF1Δ*phaC* with used gas flows (ml/min). Continuous culture was started at 64 h
